# Alzheimer’s disease pattern derived from relative cerebral flow as an alternative for the metabolic pattern using SSM/PCA

**DOI:** 10.1186/s13550-022-00909-8

**Published:** 2022-06-23

**Authors:** Débora E. Peretti, David Vállez García, Remco J. Renken, Fransje E. Reesink, Janine Doorduin, Bauke M. de Jong, Peter P. De Deyn, Rudi A. J. O. Dierckx, Ronald Boellaard

**Affiliations:** 1 Department of Nuclear Medicine and Molecular Imaging, University Medical Center Groningen, University of Groningen, Groningen, The Netherlands; 2 Department of Biomedical Sciences of Cells and Systems, Cognitive Neuroscience Center, University Medical Center Groningen, University of Groningen, Groningen, The Netherlands; 3 Department of Neurology, Alzheimer Centre, University Medical Center Groningen, University of Groningen, Groningen, The Netherlands; 4 Laboratory of Neurochemistry and Behaviour, Institute Born-Bunge, University of Antwerp, Antwerp, Belgium; 5 Department of Radiology and Nuclear Medicine, Location VU Medical Center, Amsterdam University Medical Center, De Boelelaan 1117, 1081 HV Amsterdam, The Netherlands

**Keywords:** Alzheimer’s disease, Disease pattern, Relative cerebral blood flow, SSM/PCA

## Abstract

**Background:**

2-Deoxy-2-[^18^F]fluoroglucose (FDG) PET is an important tool for the identification of Alzheimer’s disease (AD) patients through the characteristic neurodegeneration pattern that these patients present. Regional cerebral blood flow (rCBF) images derived from dynamic ^11^C-labelled Pittsburgh Compound B (PIB) have been shown to present a similar pattern as FDG. Moreover, multivariate analysis techniques, such as scaled subprofile modelling using principal component analysis (SSM/PCA), can be used to generate disease-specific patterns (DP) that may aid in the classification of subjects. Therefore, the aim of this study was to compare rCBF AD-DPs with FDG AD-DP and their respective performances. Therefore, 52 subjects were included in this study. Fifteen AD and 16 healthy control subjects were used to generate four AD-DP: one based on relative cerebral trace blood (*R*_1_), two based on time-weighted average of initial frame intervals (ePIB), and one based on FDG images. Furthermore, 21 subjects diagnosed with mild cognitive impairment were tested against these AD-DPs.

**Results:**

In general, the rCBF and FDG AD-DPs were characterized by a reduction in cortical frontal, temporal, and parietal lobes. FDG and rCBF methods presented similar score distribution.

**Conclusion:**

rCBF images may provide an alternative for FDG PET scans for the identification of AD patients through SSM/PCA.

**Supplementary Information:**

The online version contains supplementary material available at 10.1186/s13550-022-00909-8.

## Background

Alzheimer’s disease (AD) is the most common cause of dementia in the elderly population, and it is characterized by a reduction of metabolism in the parietal and temporal lobes, and, in later stages, the frontal lobe [1]. These changes can be assessed in vivo in patients with the use of 2-Deoxy-2-[^18^F]fluoroglucose (FDG) positron emission tomography (PET) imaging, a radioactive glucose analogue widely used in neuroimaging studies. Since AD affects the brain in such a particular manner, FDG PET images may help with the identification of this disease [2].

However, the most specific marker of AD is the deposition of amyloid-β (Aβ) plaques in the brain of patients [3]. These deposits can be visualized in vivo through PET radiotracers designed to bind to Aβ, such as [^11^C]-labelled Pittsburgh Compound B (PIB) [4]. Combination of FDG and PIB offers unique information for the correct classification of AD patients from other neurodegenerative diseases. Nevertheless, dual-tracer studies are more expensive, increase patient discomfort and exposure to radiation. Therefore, the use of a single-tracer PET image that could characterize more than one aspect of a disease is of practical interest.

Earlier studies have already shown that brain metabolism and regional cerebral blood flow (rCBF) are associated [5]. Since then, several studies have explored to what extent this association runs, both in general and specifically in AD [6–8]. Moreover, rCBF values derived from dynamic PIB PET scans have already been shown to be well correlated with FDG, both through parametric images of relative cerebral tracer flow (*R*_1_) and weighted average of the initial frames of the PIB scan (ePIB) [9–16]. Previous studies have shown that, despite *R*_1_ and ePIB not being the same as rCBF, they provide rCBF surrogate images that are well correlated with the gold standard measure of rCBF [10].

While most of the previous studies comparing metabolism and rCBF measures were performed based on regional uptake values [9, 17–19], not many investigations have been performed in AD population on a voxel level using rCBF images derived from dynamic PET scans [20, 21]. Scale Subprofile Modelling using Principal Component Analysis (SSM/PCA) is a network analysis technique that combines information from a group of patients and healthy volunteers to generate an image that characterizes the tracer or biomarker specific disease pattern [22, 23]. This generated pattern can then be used to test new subjects and give them a score that represents how much they express this characteristic pattern in their images. Moreover, one of the advantages of this technique is that it is independent of the user’s assessment of subject classification, which relies on the reader’s experience and can introduce variability in classification [1, 24, 25].

The aim of this study was to generate rCBF disease-related AD pattern (AD-DP) from dynamic PIB PET studies using SSM/PCA, extending a previous work using an FDG template only [10], and then compare the results with the ones obtained from FDG PET scans. To this end, *R*_1_ and ePIB images of two different (early) time intervals were generated and analysed. Then, correlations between characteristic disease patterns and subject’s scores obtained by the rCBF images were compared to those from FDG images.

## Material and methods

### Subjects, PET acquisition, and image processing

A cohort of fifty-two subjects was drawn from a large on-going study at the memory clinic of the University Medical Center Groningen (UMCG), Groningen, The Netherlands. All subjects gave their written informed consent to participate in the study. This study was approved by the Medical Ethical Committee of the UMCG (2014/320) and was conducted in agreement with the Declaration of Helsinki and subsequent revisions. Of all subjects, 15 were diagnosed as AD, 11 as Mild Cognitive Impairment due to AD (MCI+), 10 as MCI not due to AD (MCI−), and 16 healthy controls (HC) based on the National Institute on Aging and the Alzheimer’s Association Research Framework [3], and the Petersen criteria [26]. Healthy controls were recruited via advertisement, and subjects were included if they had no cognitive complaints and a mini-mental state examination (MMSE) score of 28 or above. Each subject underwent two PET scans: a static FDG and a dynamic PIB. From the FDG PET scan, a standardized uptake value normalized by cerebellar grey matter uptake (SUVR) image was generated. From the dynamic PIB PET scan, three rCBF images were generated, namely *R*_1_ by pharmacokinetic modelling of the dynamic scan (using Simplified Reference Tissue Model 2 (SRTM2) [27] and the grey matter of the cerebellum as the reference tissue), and 2 SUVR images by taking the time-weighted average of the initial frame intervals, i.e. ePIB(20–120 s) and ePIB(1–8 min). Therefore, each subject had a set of four images. A complete description of patient inclusion methods is described elsewhere [10].

*R*_1_ images were generated by applying pharmacokinetic modelling in a voxel-based level using the grey matter of the cerebellum as a reference region. The simplified reference tissue model was first applied to make an estimation of binding potential. Then, voxels with a binding potential value above 0.05 were selected and the median value of the estimated efflux parameter of the reference region was fixed. Finally, the SRTM2 was applied with a restriction on the range of the apparent efflux rate constant values, with a minimum of 0.01 and a maximum of 0.3 and 80 basis functions to generate the final *R*_1_ parametric maps. For a thorough comparison of the dynamics of *R*1 and different frame intervals of early phase PIB scans across AD, MCI, and HC groups, the authors refer the reader to a previous publication by Rodriguez-Vieitez and colleagues [15].

All images were normalized to the Montreal Neurological Institute space using tissue probability maps. Anatomical regions were defined from the Hammers atlas [28]. Regions were combined accordingly to the lobe to which they belong to. Additional File 1: Table S1 shows a complete list of regions that were combined to generate lobe volumes of interest. Table 1 presents a summary of the demographic characteristics of the included subjects in this study. Further details on tracer synthesis, PET experimental design, and image processing are described elsewhere [10, 29].

**Table 1:** Demographic characteristics of the subjects

		AD (*n* = 15)	MCI+ (*n* = 11)	MCI− (*n* = 10)	HC (*n* = 16)
*Sex*					
	Male	9	7	8	11
	Female	6	4	2	5
Age (years)		65 ± 8	65 ± 5	67 ± 9	69 ± 5
MMSE score		25 ± 3	27 ± 2	24 ± 6	30 ± 1

### Scale subprofile modelling using principal component analysis

SSM/PCA was applied using an in-house software based on the work of Spetsieris and colleagues [22] adapted for the use of quantitative images derived from pharmacokinetic modelling and intensity normalized images (SUVR) [20]. In summary, spatially normalized images of 15 AD patients and 16 HC subjects were masked so that only brain data were further used in the analysis. Images were converted into a matrix where each subject corresponds to a column and each row is a voxel in the image. Data were centred per subject and then an average HC image was generated. This image was subtracted from all subject’s images. PCA was applied to the data, and components were ordered by explained variance. The first components that, when combined, explained at least 50% of data variance were selected. Lastly, a stepwise forward logistic regression was performed to generate a pattern. The Akaike information criterion was used to define the best image that represents the AD-DP. A leave-one-out cross-validation (LOOCV) approach was used to verify the stability of the AD-DP scores. For each subject that was left out, a new AD-DP was generated using the remaining subjects and the left-out subject received a new score based on this AD-DP. All subject’s image received a score by the inner product of the image against the AD-DP. For the subjects that were used to generate the AD-DP, the LOOCV score was taken to reduce bias. Finally, the scores were standardized to a *Z*-score using the mean standard deviation of the LOOCV HC group. Average and standard deviation values for all regions included in the Hammers atlas and the larger lobe volumes of interest were extracted from each AD-DP.

### Comparison between disease patterns

Pearson’s correlation coefficients were calculated comparing all AD-DPs. To further explore the differences and similarities between the rCBF AD-DPs and the FDG AD-DP, joint histograms were plotted. A joint histogram is a multidimensional histogram created from a set of voxel values with the same location in the brain. The *x*- and *y*-axes represent voxel values in the AD-DPs generated from different images (i.e. FDG and rCBF). A voxel in the FDG AD-DP (*x*-axis) with a specific value might have a different value in the rCBF-generated DP (*y*-axis). The joint histogram contains the counts of how many voxels have the same combination of values, which were plotted in a base 10 logarithmic amplitude scale. To better quantify these relationships, a linear regression was used to explore the correlations. In these comparisons, the FDG AD-DP was considered the independent variable, while the rCBF patterns the dependent, as it was done before for the scores. This configuration allows for an investigation of how the regional cerebral blood flow is explained by the metabolism. A *p* value of 0.05 was used as a significance threshold for all evaluations.

### Statistical analysis of scores

Receiver operating characteristic (ROC) curves were generated using the scores from AD and HC groups to find the optimal threshold for classifying subjects based on Youden’s method [30]. Confidence intervals (CI) for the area under the curve were calculated using a 95% interval. To avoid a possible bias of using the same scores from the subjects that were used to generate the disease pattern, this analysis was performed using only the LOOCV scores.

An ANOVA per image type was performed to test if scores from different groups of subjects were significantly different from each other. The *p* values were then corrected for multiple comparisons using Tukey’s approach.

A general linear model was used to explore the relationship between the scores from the rCBF AD-DPs (dependent variable) and the ones from the FDG AD-DP (independent variable) for all subjects. Pearson correlation coefficients were also computed to explore the interrelationship between metabolic and rCBF scores. Furthermore, a Bland–Altman plot was used to evaluate the agreement between metabolic and rCBF scores [31, 32]. The agreement interval was calculated as 1.96 × standard deviation. Moreover, linear regressions were made to assess the bias of each rCBF score compared to the FDG scores. All results were analysed using RStudio (version 1.2.5033, R version 3.6.3).

## Results

### Description of disease patterns

**Fig. 1:**
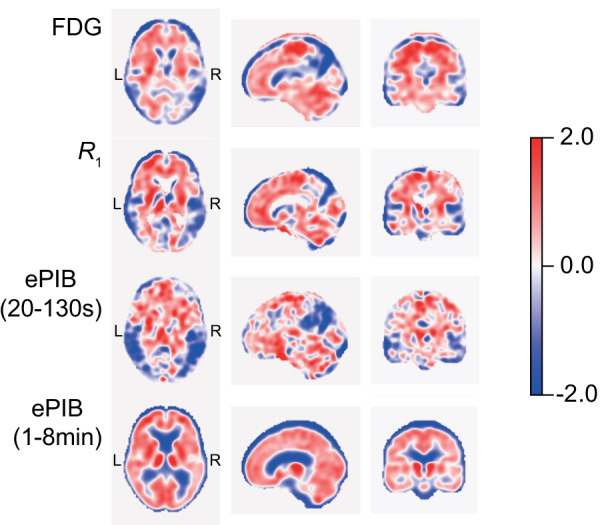
Disease Patterns. AD-DPs results from the comparison between HC and AD patients for FDG (first row), *R*_1_ (second row), ePIB(20–130 s) (third row), and ePIB(1–8 min) (fourth row). Blue and red colours indicate negative and positive voxel values, respectively. The closer to white, the closer the voxel value is to zero. All colour scales were adjusted to the same range

Overall, the FDG, *R*_1_, and ePIB(20–130 s) AD-DP (Fig. 1) agreed with the expected AD patterns of previous studies, presenting a general cortical decreased metabolism in patients when compared to HC subjects, with the parietal lobe showing the largest relative reduction in metabolism and rCBF. This can be seen by the negative voxels depicted in blue in Fig. 1. However, the ePIB(1–8 min) images resulted in an AD-DP that resembles more a pattern of amyloid deposition [20] than the one expected for metabolism or rCBF. This pattern was characterized by a generalized relative increase in signal (positive voxels depicted in red in Fig. 1) in grey matter of AD patients when compared to HC subjects. Table 2 contains the details of the principal components used to generate the final disease patterns, and Table 3 shows the average and standard deviation values of larger brain lobes for each AD-DP. Additional file 1: Table S1 contains average and standard deviation values for all regions included in the Hammers atlas.

**Table 2:** Composition of principal components (PC) in the disease pattern (DP) of each imaging method

	Included PCs	PCs in DP	Variance explained by DP (%)
FDG	5	1	24.2
*R*_1_	6	1, 2	30.5
ePIB(20–130 s)	7	1, 2, 3	33.8
ePIB(1–8 min)	4	1	29.1

**Table 3:** Volume, average, and standard deviation values of the AD-DPs for larger brain regions

Region	Volume (cm^3^)	FDG	*R*_1_	ePIB(20–130 s)	ePIB(1–8 min)
Brainstem	29.29	0.66 ± 0.33	0.41 ± 0.94	0.39 ± 0.63	0.19 ± 0.92
Frontal lobe	501.06	0.07 ± 1.05	0.05 ± 1.02	0.23 ± 0.77	0.26 ± 0.89
Occipital lobe	172.99	− 0.25 ± 0.76	− 0.11 ± 0.65	0.50 ± 0.79	0.36 ± 0.71
Parietal lobe	307.86	− 0.58 ± 1.23	− 0.41 ± 0.95	− 0.68 ± 1.01	0.00 ± 0.82
Temporal lobe	285.69	− 0.11 ± 0.81	− 0.23 ± 0.82	− 0.45 ± 0.84	0.03 ± 0.68
Cerebellum	180.89	0.73 ± 0.65	0.58 ± 0.89	0.77 ± 1.00	0.44 ± 0.83
Posterior cingulate cortex	6.38	− 1.30 ± 1.17	− 0.76 ± 0.91	− 1.16 ± 0.91	0.33 ± 0.82

### Correlations and joint histograms

When compared to the FDG AD-DP, rCBF patterns resulted in the following correlations: *R*_1_ 0.79, ePIB(20–130 s) 0.59, and ePIB(1–8 min) 0.35. When compared between each other, rCBF AD-DPs presented lower correlation values: *R*_1_ and ePIB(20–130 s) 0.62, *R*_1_ and ePIB(1–8 min) 0.30, and ePIB(20–130 s) and ePIB(1–8 min) 0.02.

**Fig. 2:**
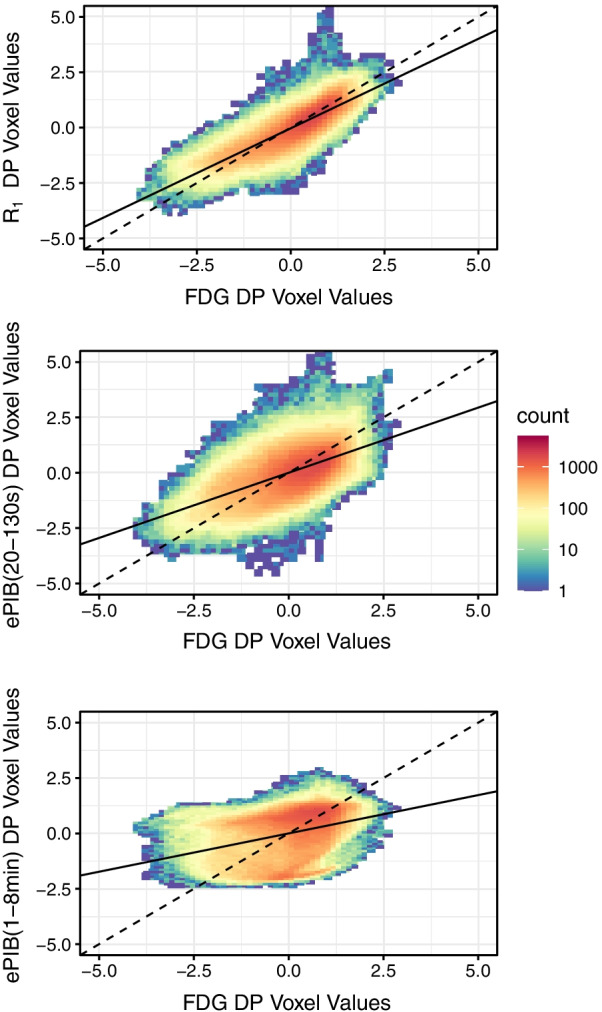
Joint Histograms of the disease patterns*.* Joint histograms of the FDG and R1 (top), ePIB(20–130 s) (middle), and ePIB(1–8 min) (bottom) DPs. The dashed line corresponds to the identity, and the solid line, to the linear regression of the data from the DPs. The bin counts are displayed in base 10 logarithmic amplitude scale

Figure 2 shows the joint histograms comparing the FDG AD-DP with either the *R*_1_ (Fig. 2, top), the ePIB(20–130 s) (Fig. 2, middle), or the ePIB(1–8 min) (Fig. 2, bottom) DPs. These results suggest a high correlation between *R*_1_ and FDG AD-DPs, with FDG metabolism accounting for 69% of variability (*R*^2^ = 0.69, *p* < 0.01, slope = 0.81, intercept = − 0.03). Meanwhile, the FDG metabolic pattern accounted for only 35% of the variability of the ePIB(20–130 s) AD-DP (*R*^2^ = 0.35, *p* < 0.01, slope = 0.59, intercept = 0) and 12% of the variance of the ePIB(1–8 min) AD-DP (*R*^2^ = 0.12, *p* < 0.01, slope = 0.35, intercept = 0).

### ROC curves

**Fig. 3:**
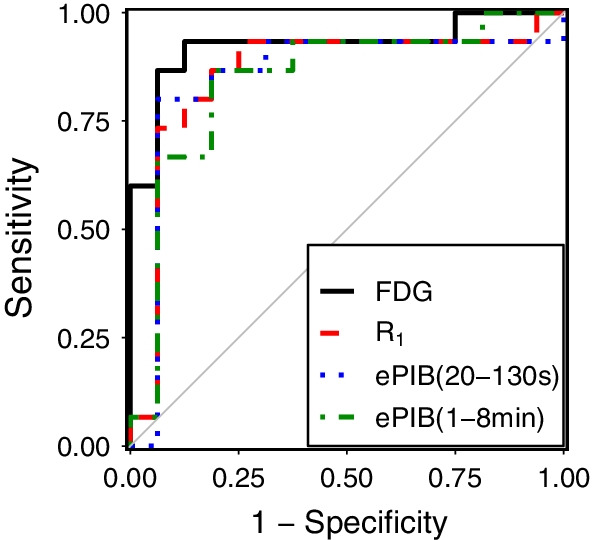
ROC Plots*.* ROC plot with the curves of FDG (solid black line, AUC = 0.93), *R*_1_ (dashed red, AUC = 0.86), ePIB(20–130 s) (dotted blue, AUC = 0.69), and ePIB(1–8 min) (dot-dashed green, AUC = 0.85)

Figure 3 shows the ROC curves used to define the threshold that classifies subjects as AD patients or HC. For the FDG images, the optimal threshold found was 1.1 with an area under the curve (AUC) of 0.93 (CI 0.82–1.0). Meanwhile, all rCBF methods resulted in a similar AUC. *R*_1_ presented an AUC, of 0.86 (CI 0.7–1.0), with a threshold for classification of 0.35. Then, ePIB(20–130 s) had a threshold of 0.69 and an AUC of 0.85 (CI 0.68–1.0). Finally, ePIB(1–8 min) resulted in a threshold of 0.58 and an AUC of 0.85 (CI 0.69–0.99).

### Distribution of scores

**Fig. 4:**
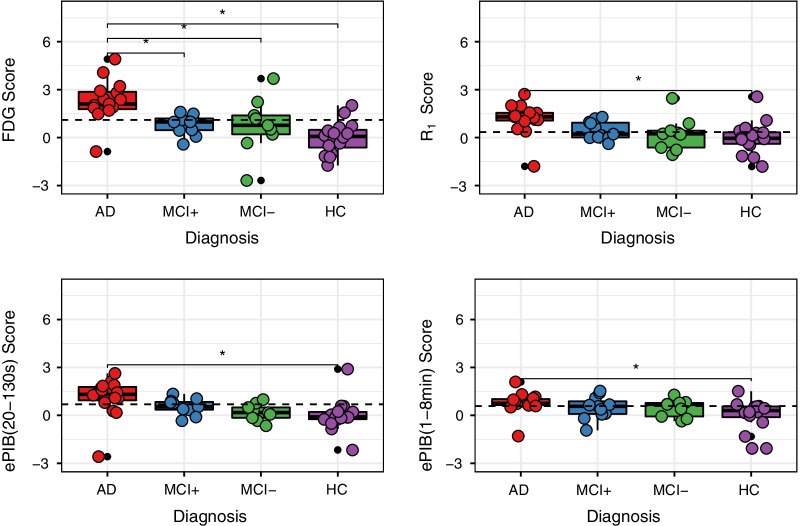
Distribution of Scores per Method*.* Distribution of the subjects’ normalized scores from FDG (top left), *R*_1_ (top right), ePIB(20–130 s) (bottom left), and ePIB(1–8 min) (bottom right). Boxes represent the interquartile range of distribution, with the median per group showing as a full line, the whiskers expanding up to 1.5 times the interquartile range, and the outliers are presented as black dots. Coloured circles represent individual subjects’ scores within the groups. AD patients are depicted in red, MCI+ , in blue; MCI−, in green; and HC subjects, in purple. The dashed lines correspond to the threshold for classifying subjects as AD patients, and the stars represent the differences between groups that are statistically significant

Figure 4 depicts the distribution of scores for each subject for all methods used in this analysis. In general, the AD group of patients showed a higher score than the other groups, followed by the MCI+ subjects. The resemblance between group scores distributions of rCBF and FDG methods was also notable.

In general, all image methods presented a statistically significant different distribution of scores between groups (ANOVA, *p* < 0.05). After correction for multiple comparisons, FDG scores (Fig. 4, top left) were significantly different between AD and MCI+ (difference between groups: 1.48 ± 0.47, *p* = 0.02), MCI− (1.53 ± 0.50, *p* = 0.02), and HC (2.31 ± 0.43, *p* < 0.01) groups. Meanwhile, the rCBF methods only presented statistically significant differences between the AD and HC groups. *R*_1_ (Fig. 4, top right) showed a difference of means of 1.16 ± 0.34 (*p* < 0.01); ePIB(20–13 s) (Fig. 4, bottom left) of 1.11 ± 0.33 (*p* < 0.01); and ePIB(1–8 min) (Fig. 4, bottom right), 0.77 ± 0.28 (*p* = 0.04). However, a trend was found in *R*_1_ (0.94 ± 0.39, *p* < 0.1) and ePIB(20–130 s) (0.92 ± 0.39, *p* < 0.1) scores for the differentiation between AD and MCI− subjects. Mean, standard deviation, standard error of the mean, and range of scores for all groups of subjects for each method can be seen in Additional file 2: Table S2.

### Correlation between rCBF and metabolism scores

**Fig. 5:**
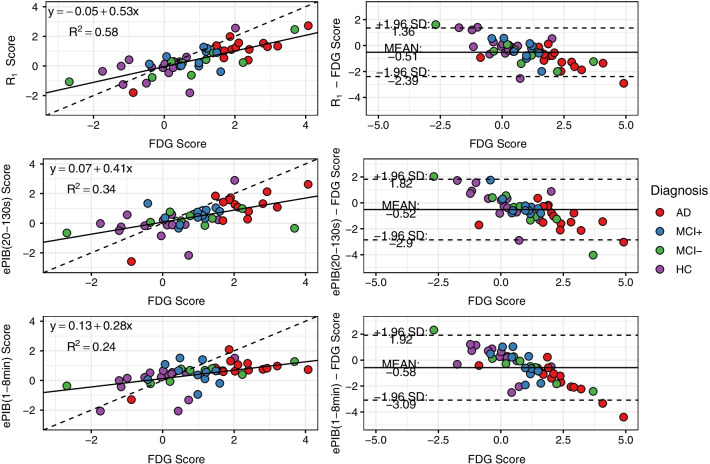
Scatter and Bland–Altman Plots*.* Scatter plots (left column) of scores from *R*_1_ parametric images (top), ePIB(20–130 s) (middle), and ePIB(1–8 min) (bottom) images (*y*-axis), compared to FDG (*x*-axis). The dashed line represents the identity. Results from the linear regression applied to the data are shown in the upper left corner of the plots and as a solid line. Bland–Altman plots (right column) show the difference between the scores provided by *R*_1_ (top), ePIB(20–130 s) (middle), and ePIB(1–8 min) (bottom) and FDG. The solid line is at the average difference between the scores, and the dashed lines delimit the limits of agreement of the 95% interval (at mean ± 1.96 × standard deviation). Data points are coloured according to group: in red, the AD patients; in blue, the MCI+ ; in green, the MCI−; and in purple, the HC subjects

The scatter plots presented in the left column of Fig. 5 suggest a moderate correlation between the FDG and *R*_1_ scores, and a low correlation between FDG and the other rCBF methods. FDG scores were moderately predictive of the *R*_1_ scores (*R*^2^ = 0.58, *p* < 0.01, slope = 0.53, intercept = − 0.05), while ePIB(20–130 s) scores presented a weak correlation (*R*^2^ = 0.34, *p* < 0.01, slope = 0.41, intercept = 0.07), and ePIB(1–8 min) showed the smallest correlation (*R*^*2*^ = 0.24, *p* < 0.01, slope = 0.28, intercept = 0.13).

### Bias assessment

The bias found between the FDG and rCBF scores presented a linear relationship (Fig. 5, right column): while the lower scores were overestimated, the higher rCBF scores were underestimated when compared to FDG. Linear regressions of Bland–Altman plots resulted in a slope of − 0.47 and an intercept of − 0.05 for *R*_1_; − 0.59 and 0.07 for ePIB(20–130 s); and − 0.72 and 0.13 for ePIB(1–8 min) [31, 32].

## Discussion

This study aimed to investigate the use of rCBF images derived from dynamic PIB PET scans as an alternative to FDG through an SSM/PCA analysis for classifying AD patients versus HC subjects. The metabolic pattern generated by FDG images has been identified as an appropriate tool to identify AD patients [2, 20, 23]. Due to the correlation between metabolism and blood flow [33], a similar characteristic AD pattern was expected from rCBF images [20]. Furthermore, rCBF measures have been shown to correlate, at least partially, with cognitive impairment in AD patients [34], increasing the importance of these images in AD classification. Therefore, the use of amyloid-derived rCBF images as a proxy for an FDG scan is an attractive alternative that may reduce study costs, decrease patient discomfort, and minimize radiation exposure because both rCBF and specific binding information can be driven from a single dynamic PET scan.

The generated DPs presented a cortical decrease in flow [*R*_1_ and ePIB(20–130 s)] and metabolism (FDG) in AD patients when compared to HC subjects. As previously mentioned, this similarity between patterns was expected due to the existent relationship between metabolism and blood flow [5, 9] and due to previous studies using voxel-based univariate analysis of the images [9]. Still, some differences between the patterns were found in regions that are already known to be hyperperfused, which is consistent with previously published results [9, 20, 35]. However, the most interesting point to observe from this analysis is the difference in pattern from the ePIB(1–8 min) as compared to the other methods (Fig. 1). This time interval between 1 and 8 min has been recommended as the time interval with the best visual correlation with FDG, based on the correlation of its regional values and those of FDG images [16]. However, the generated AD-DP in this study shows a pattern more closely related to the amyloid deposition pattern of AD patients, which showed increased signal in grey matter cortical regions in scans acquired later during the scan [20]. Furthermore, a previously published SPM voxel-based analysis of the same images showed that this time interval was also not able to differentiate between patients and controls [9]. This result is consistent with the hypothesis that this time interval is too long, and the signal is already affected by Aβ binding, resulting in an image that does not reflect purely rCBF [9], Therefore, for the remaining of this discussion, ePIB(1–8 min) data will no longer be addressed.

The distribution presented in Fig. 4 showed that all methods presented significantly different scores between the AD and HC groups. However, only FDG was capable of distinguishing the group of AD patients from MCI+ and MCI− subjects. Even though MCI + is also known as ‘MCI due to Alzheimer’, no method was able to generate subject scores that were statistically different between MCI+ and MCI− or HC groups. This could be due to the fact that rCBF images are less sensitive to subtle changes than metabolic scans [9] and, therefore, metabolism images might be able to capture subtle changes that are not reflected by rCBF. However, a larger dataset of patients might increase the stability of the DP, which may allow for these subtler changes to be captured. Furthermore, an independent testing group of AD and HC subjects could better assess the accuracy of the generated AD-DP. Other automated methods for image assessment for AD classification have shown to be more sensitive to assess disease progression of MCI patients [1, 36]. Moreover, the same set of subjects used in this study were previously analysed using an automated tool for assessment of AD, which resulted in a good contrast between AD patients and HC subjects but could not distinguish between MCI subjects as well [10]. Therefore, these patients were added to this analysis to evaluate the use of the SSM/PCA technique to evaluate their AD-DP expression.

Moreover, Fig. 4, in combination with Fig. 5, shows a smaller range of rCBF scores when compared to metabolism. This suggests that the reduction in FDG uptake in AD patients is greater than the reduction in *R*_1_ and ePIB(20–130 s) when compared to HC subjects. Therefore, the AD-DP expression through the subject scores results in smaller values for rCBF methods and ensues a greater bias for larger scores when compared with FDG. Moreover, the more extensive range of scores shown in Fig. 4, the higher AUC, and the largest correlation to metabolism scores indicate that *R*_1_ might be the most suited rCBF method for generating an AD-DP through SSM/PCA as a proxy for FDG scans. In addition, *R*_1_ images provide a pure measure of relative radiotracer flow due to the inherent quantitative aspect of parametric images, while SUVR shows a mixture of specific and non-specific binding, free tracer in tissue, and blood signal, which might have affected the ePIB(20–130 s) performance for subject identification [37]. Finally, Fig. 5 shows that the bias on rCBF scores when compared to FDG is negative for higher scores and positive for lower scores, which further indicates that rCBF scores are not as robust as the FDG scores when differentiating between AD and HC. Further studies using other techniques for estimating rCBF images are worthy of exploration, as some of them might improve results even for MCI subjects. However, as SUVR is the most frequently used approach in clinical research due to its simplicity, it was the one chosen for this study.

Although the results presented in the previous section suggest a good correlation of AD-DPs and subject scores between rCBF and FDG, it is important to mention that, from a physiological point of view, there is no perfect equivalence between them. However, these results sustain the use of SSM/PCA as a classification technique to be used not only with FDG PET scans and AD, but also for other types of images and diseases. Furthermore, this analysis was performed using PIB as a radiotracer, but similar results can be expected for other ^18^F-labelled amyloid radiotracers such as [^18^F]Florbetapir, [^18^F]Florbetaben, and [^18^F]Flutemetamol [38]. Yet, further research is necessary to confirm this. Moreover, this study was performed on a relatively small sample of subjects. Larger cohorts might yield more accurate results by providing a more stable pattern, which might find statistically significant differences where this study found only a trend, such as a significant difference between AD and MCI+ patients. In addition, longitudinal datasets might be useful for analysing the efficiency of SSM/PCA in predicting the conversion of MCI subjects to AD. Finally, SSM/PCA might be an interesting technique to be further explored for the identification of patients both in the clinic and in research settings, since it allows for testing of new subjects that are not related to the ones used to generate the DP.

Amyloid PET imaging is already used in clinical settings for the assessment of deposits to identify AD patients. A dynamic scan may provide rCBF images that, in some cases, might be enough for an assessment on neurodegeneration, dismissing the need of a second FDG PET scan for this analysis. Performing a single scan reduces patient burden and exposure to radiation, and costs in terms of FDG production and scanner time. Furthermore, in a clinical setting, the assessment of PET images is mostly performed visually by an expert neuroradiologist or neurologist. Previous studies have found that visual assessment relies on the reader’s experience and even then, the agreement between the readers is not always optimal [39]. An automated technique such as the SSM/PCA has the potential of resulting in more consistent and accurate subject assessment. Moreover, by providing scores that quantify the expression of the DP in a subject, this technique may be used for classifying subjects in stages. Finally, SSM/PCA allows for the generation of a centre-specific DP that can be generated with both FDG and rCBF images. This enables the comparison of rCBF images with more than just an FDG pre-set database that is available in software packages available for the clinic. However, the technique proposed in this study requires a local dataset of patients and controls for the generation of the disease characteristic patterns, as this technique has been shown to be sensitive to scanner and reconstruction effects [40], which may not be available in all imaging centres.

While rCBF images might be used as a surrogate for FDG PET in more extreme cases, it has been shown not to fully capture the smaller changes in patients when compared to controls. This is a key point when identifying patients in a preclinical phase, for example. Amyloid imaging is the first recommended imaging modality (after structural imaging) in cases of suspected AD diagnosis [41]. However, in cases where amyloid imaging is inconclusive, to exclude possible amyloid positive co-pathologies, to make a short-term prognosis, or to make a better assessment of the stage of the disease, FDG PET is still recommended. By performing a dynamic scan instead of a static one, it is possible to generate rCBF from the same scan, avoiding putting the subject through the burden of a second PET scan.

## Conclusion

The aim of this study was to explore whether rCBF images derived from dynamic PIB PET scans can be used as an alternative for an FDG PET scan for the identification of AD patients using SSM/PCA as the image analysis technique. From the different approaches to generate the rCBF images, *R*_1_ parametric maps have shown the best correlation with FDG and the best classification ability between groups. However, the sensitivity of *R*_1_ AD-DP scores is not as great as FDG scores. This suggests that *R*_1_ parametric maps can be an alternative for an FDG PET scan for diagnostic purposes when using SSM/PCA as an initial assessment of AD patients. A second FDG PET scan might be necessary in more initial stages of neurodegeneration or to differentiate between other disorders.

## Supplementary Information

**Additional file 1**. **Table S1**: Average and standard deviation values if the AD-DPs for brain regions included in the Hammers atlas.

**Additional file 2**. **Table S2**: Number of subjects (*N*), mean, standard deviation (SD), standard error of the mean (SEM), and range of scores per diagnosis for each type of image used in the analysis.

## Data Availability

The datasets generated and/or analysed during the current study are available with the corresponding author upon reasonable request.

## Abbreviations

AD: Alzheimer’s disease
FDG: 2-Deoxy-2-[^18^F]fluoroglucose
PET: Positron emission tomography
Aβ: Amyloid-β
PIB: [^11^C]-labelled Pittsburgh Compound B
rCBF: Regional cerebral blood flow
*R*_1_: Relative cerebral tracer flow
ePIB: Initial frames of the PIB scan
SSM/PCA: Scaled subprofile modelling using principal component analysis
UMCG: University Medical Center Groningen
MCI+: Mild cognitive impairment due to AD
MCI−: Mild cognitive impairment not due to AD
HC: Healthy control
SUVR: Standardized uptake value normalized by cerebellar uptake
AD-DP: Disease-related Alzheimer’s disease pattern
LOOCV: Leave-one-out cross-validation
ROC: Receiver operating characteristic
PC: Principal component
DP: Disease pattern
AUC: Area under the curve
SRTM2: Simplified reference tissue model 2
MMSE: Mini-mental state examination
