# Supplementary material for: Alzheimer’s disease pattern derived from relative cerebral flow as an alternative for the metabolic pattern using SSM/PCA
Source: EJNMMI Res. 2022 Jun 23;12:37. doi: 10.1186/s13550-022-00909-8 (PMC9226207; doi:10.1186/s13550-022-00909-8)
Supplement: Supplementary file 1 — Additional file 1. Table S1: Average and standard deviation values if the AD-DPs for brain regions included in the Hammers atlas. [file 13550_2022_909_MOESM1_ESM.docx]

**Supplementary Table S1.** Average and standard deviation values if the AD-DPs for brain regions included in the Hammers atlas.

| **Region** | **Lobe** | **FDG** | **R1** | **ePIB(20-130s)** | **ePIB(1-8min)** |
| --- | --- | --- | --- | --- | --- |
| Middle frontal gyrus L | Frontal Lobe | -0.85 ± 0.92 | -0.46 ± 0.87 | -0.17 ± 0.69 | 0.26 ± 1.02 |
| Middle frontal gyrus R |  | -0.68 ± 1.15 | -0.49 ± 1.33 | -0.40 ± 0.73 | 0.48 ± 0.96 |
| Precentral gyrus L |  | 0.61 ± 0.77 | 0.33 ± 0.65 | 0.48 ± 0.78 | 0.34 ± 0.85 |
| Precentral gyrus R |  | 0.46 ± 1.00 | 0.08 ± 0.96 | 0.29 ± 0.73 | 0.38 ± 0.90 |
| Straight gyrus L |  | 0.29 ± 0.36 | 0.20 ± 0.64 | 0.55 ± 0.80 | 0.82 ± 0.38 |
| Straight gyrus R |  | 0.51 ± 0.33 | 0.29 ± 0.50 | 0.66 ± 0.53 | 0.94 ± 0.36 |
| Anterior orbital gyrus L |  | 0.01 ± 0.69 | 0.00 ± 0.99 | 0.12 ± 0.66 | 0.82 ± 0.61 |
| Anterior orbital gyrus R |  | 0.44 ± 0.59 | 0.50 ± 1.04 | 0.01 ± 0.60 | 0.91 ± 0.59 |
| Inferior frontal gyrus L |  | -0.39 ± 0.64 | -0.43 ± 0.70 | -0.02 ± 0.76 | 0.22 ± 0.71 |
| Inferior frontal gyrus R |  | 0.13 ± 0.77 | -0.07 ± 1.00 | 0.10 ± 0.74 | 0.75 ± 0.59 |
| Superior frontal gyrus L |  | -0.25 ± 0.98 | 0.17 ± 0.74 | 0.42 ± 0.73 | 0.28 ± 0.85 |
| Superior frontal gyrus R |  | -0.02 ± 0.96 | -0.09 ± 0.97 | 0.54 ± 0.82 | 0.43 ± 0.84 |
| Medial orbital gyrus L |  | 0.10 ± 0.50 | -0.11 ± 0.84 | 0.30 ± 0.63 | 0.54 ± 0.54 |
| Medial orbital gyrus R |  | 0.40 ± 0.43 | 0.10 ± 0.73 | 0.40 ± 0.61 | 0.70 ± 0.47 |
| Lateral orbital gyrus L |  | -0.26 ± 0.67 | -0.57 ± 0.81 | -0.29 ± 0.75 | 0.40 ± 0.77 |
| Lateral orbital gyrus R |  | 0.24 ± 0.76 | -0.11 ± 1.11 | -0.08 ± 0.47 | 0.79 ± 0.73 |
| Posterior orbital gyrus L |  | 0.30 ± 0.41 | 0.21 ± 0.71 | 0.51 ± 0.67 | 0.75 ± 0.48 |
| Posterior orbital gyrus R |  | 0.65 ± 0.39 | 0.44 ± 0.79 | 0.44 ± 0.47 | 0.88 ± 0.47 |
| Subgenual anterior cingulate gyrus L |  | 0.84 ± 0.34 | 1.25 ± 0.47 | 1.28 ± 0.54 | 0.66 ± 0.54 |
| Subgenual anterior cingulate gyrus R |  | 0.75 ± 0.12 | 0.97 ± 0.37 | 0.88 ± 0.41 | 0.60 ± 0.48 |
| Subcallosal area L |  | 0.34 ± 0.20 | 0.54 ± 0.15 | 2.21 ± 0.49 | 0.19 ± 0.48 |
| Subcallosal area R |  | 0.63 ± 0.16 | 0.81 ± 0.15 | 1.57 ± 0.27 | 0.07 ± 0.42 |
| Pre-subgenual anterior cingulate gyrus L |  | 0.84 ± 0.32 | 1.12 ± 0.55 | 0.72 ± 0.43 | 1.09 ± 0.33 |
| Pre-subgenual anterior cingulate gyrus R |  | 0.82 ± 0.09 | 1.13 ± 0.26 | 1.10 ± 0.16 | 1.12 ± 0.28 |
| Hippocampus R | Temporal Lobe | 0.54 ± 0.35 | 0.26 ± 0.50 | -0.07 ± 0.34 | 0.17 ± 0.31 |
| Hippocampus L |  | 0.34 ± 0.26 | 0.18 ± 0.28 | 0.26 ± 0.40 | 0.00 ± 0.34 |
| Amygdala R |  | 0.57 ± 0.14 | 0.32 ± 0.26 | -0.10 ± 0.55 | 0.15 ± 0.29 |
| Amygdala L |  | 0.37 ± 0.22 | 0.25 ± 0.43 | 0.13 ± 0.66 | 0.14 ± 0.29 |
| Anterior temporal lobe, medial part R |  | 0.70 ± 0.47 | 0.16 ± 0.94 | 0.00 ± 0.66 | -0.01 ± 0.56 |
| Anterior temporal lobe, medial part L |  | 0.42 ± 0.53 | 0.07 ± 0.86 | 0.25 ± 0.69 | -0.02 ± 0.51 |
| Anterior temporal lobe, lateral part R |  | 0.36 ± 0.51 | -0.28 ± 0.98 | -0.19 ± 0.45 | 0.18 ± 0.63 |
| Anterior temporal lobe, lateral part L |  | 0.05 ± 0.43 | -0.47 ± 0.71 | 0.01 ± 0.39 | -0.12 ± 0.62 |
| Parahippocampal and ambient gyri R |  | 0.63 ± 0.25 | 0.50 ± 0.32 | 0.36 ± 0.52 | 0.08 ± 0.44 |
| Parahippocampal and ambient gyri L |  | 0.31 ± 0.36 | 0.17 ± 0.39 | 0.29 ± 0.65 | 0.03 ± 0.41 |
| Superior temporal gyrus, posterior part R |  | 0.10 ± 0.61 | -0.43 ± 0.87 | -0.82 ± 0.59 | 0.40 ± 0.57 |
| Superior temporal gyrus, posterior part L |  | -0.13 ± 0.50 | -0.65 ± 0.59 | -0.75 ± 0.73 | 0.15 ± 0.51 |
| Middle and inferior temporal gyrus R |  | -0.28 ± 0.82 | -0.47 ± 1.01 | -0.67 ± 0.68 | 0.30 ± 0.62 |
| Middle and inferior temporal gyrus L |  | -0.69 ± 0.78 | -0.72 ± 0.76 | -0.75 ± 0.73 | -0.05 ± 0.71 |
| Fusiform gyrus R |  | 0.67 ± 0.32 | 0.38 ± 0.47 | 0.26 ± 0.54 | 0.26 ± 0.44 |
| Fusiform gyrus L |  | 0.21 ± 0.46 | -0.16 ± 0.13 | -0.11 ± 0.78 | 0.09 ± 0.55 |
| Posterior temporal lobe L |  | -0.76 ± 0.84 | -0.48 ± 0.78 | -0.83 ± 1.05 | 0.08 ± 0.68 |
| Posterior temporal lobe R |  | -0.47 ± 0.81 | -0.42 ± 0.84 | -0.80 ± 0.88 | 0.35 ± 0.59 |
| Superior temporal gyrus, anterior part L |  | 0.48 ± 0.30 | -0.12 ± 0.39 | 0.17 ± 0.54 | -0.16 ± 0.60 |
| Superior temporal gyrus, anterior part R |  | 0.67 ± 0.45 | -0.09 ± 0.80 | -0.29 ± 0.48 | 0.05 ± 0.51 |
| Postcentral gyrus L | Parietal Lobe | 0.28 ± 0.74 | 0.14 ± 0.75 | 0.20 ± 0.68 | 0.13 ± 0.85 |
| Postcentral gyrus R |  | 0.46 ± 0.84 | 0.09 ± 0.71 | 0.26 ± 0.62 | 0.42 ± 0.78 |
| Superior parietal gyrus L |  | -1.31 ± 1.00 | -0.98 ± 0.90 | -1.21 ± 0.83 | 0.28 ± 0.78 |
| Superior parietal gyrus R |  | 1.50 ± 1.05 | -1.02 ± 0.84 | -1.10 ± 0.99 | 0.30 ± 0.78 |
| Inferiolateral remainder of parietal lobe L |  | -1.52 ± 1.04 | -0.83 ± 0.76 | -1.28 ± 0.83 | -0.24 ± 0.73 |
| Inferiolateral remainder of parietal lobe R |  | -1.26 ± 1.15 | -0.80 ± 0.93 | -1.16 ± 0.82 | 0.10 ± 0.70 |
| Lateral remainder of occipital lobe L | Occipital Lobe | -0.77 ± 0.68 | -0.21 ± 0.65 | -0.78 ± 0.72 | 0.14 ± 0.66 |
| Lateral remainder of occipital lobe R |  | -0.40 ± 0.75 | -0.19 ± 0.60 | -0.66 ± 0.78 | 0.33 ± 0.64 |
| Lingual gyrus L |  | 0.12 ± 0.56 | 0.02 ± 0.72 | -0.25 ± 0.72 | 0.85 ± 0.43 |
| Lingual gyrus R |  | 0.42 ± 0.49 | -0.07 ± 0.65 | -0.01 ± 0.80 | 0.99 ± 0.34 |
| Cuneus L |  | -0.21 ± 0.47 | -0.12 ± 0.69 | -0.11 ± 0.58 | 0.76 ± 0.57 |
| Cuneus R |  | -0.13 ± 0.51 | -0.34 ± 0.59 | -0.42 ± 0.69 | 0.84 ± 0.49 |
| Caudate nucleus L | Central Structures | -0.10 ± 0.50 | -0.07 ± 0.66 | 1.33 ± 0.59 | 0.21 ± 0.86 |
| Caudate nucleus R |  | 0.03 ± 0.48 | 0.30 ± 0.71 | 1.35 ± 0.61 | -0.14 ± 0.81 |
| Nucleus accumbens L |  | 0.77 ± 0.23 | 0.75 ± 0.23 | 1.72 ± 0.28 | 1.07 ± 0.42 |
| Nucleus accumbens R |  | 1.06 ± 0.19 | 1.25 ± 0.32 | 1.82 ± 0.31 | 0.80 ± 0.41 |
| Putamen L |  | 0.92 ± 0.26 | 0.83 ± 0.61 | 0.72 ± 0.57 | 1.60 ± 0.44 |
| Putamen R |  | 1.17 ± 0.32 | 1.12 ± 0.81 | 0.82 ± 0.67 | 1.74 ± 0.43 |
| Thalamus L |  | -0.08 ± 0.69 | 0.09 ± 1.03 | 0.26 ± 0.89 | 0.72 ± 0.89 |
| Thalamus R |  | 0.16 ± 0.50 | 0.61 ± 1.09 | 0.63 ± 0.93 | 0.83 ± 0.94 |
| Pallidum L |  | 0.89 ± 0.11 | 0.95 ± 0.21 | 0.39 ± 0.34 | 1.14 ± 0.48 |
| Pallidum R |  | 1.24 ± 0.22 | 1.35 ± 0.48 | 0.49 ± 0.35 | 1.03 ± 0.41 |
| Corpus callosum |  | 0.19 ± 0.46 | 0.28 ± 0.63 | 0.05 ± 0.87 | -1.07 ± 0.51 |
| Substantia nigra L |  | 0.25 ± 0.25 | -0.34 ± 0.63 | -0.15 ± 0.29 | -0.04 ± 0.37 |
| Substantia nigra R |  | 0.32 ± 0.10 | -0.49 ± 0.22 | 0.20 ± 0.18 | -0.04 ± 0.36 |
| Insula L | Insula and Cingulate Gyri | 0.65 ± 0.37 | 0.36 ± 0.57 | 0.65 ± 0.59 | 0.56 ± 0.45 |
| Insula R |  | 0.81 ± 0.30 | 0.56 ± 0.61 | 0.54 ± 0.48 | 0.83 ± 0.45 |
| Cingulate gyrus, anterior part L |  | 0.72 ± 0.38 | 0.73 ± 0.68 | 0.45 ± 0.63 | 0.79 ± 0.41 |
| Cingulate gyrus, anterior part R |  | 0.84 ± 0.37 | 0.83 ± 0.60 | 0.95 ± 0.56 | 0.92 ± 0.41 |
| Cingulate gyrus, posterior part L |  | -0.75 ± 1.08 | -0.27 ± 0.83 | -0.94 ± 0.92 | 0.80 ± 0.51 |
| Cingulate gyrus, posterior part R |  | -0.92 ± 1.25 | -0.82 ± 0.88 | -0.91 ± 1.25 | 0.94 ± 0.43 |
| Cerebellum L | Posterior Fossa | 0.78 ± 0.62 | 0.61 ± 0.93 | 0.65 ± 1.049 | 0.61 ± 0.76 |
| Cerebellum R |  | 0.66 ± 0.67 | 0.60 ± 0.93 | 0.83 ± 0.93 | 0.61 ± 0.79 |
| Brainstem |  | 0.67 ± 0.32 | 0.44 ± 0.94 | 0.40 ± 0.62 | 0.29 ± 0.87 |
| White matter L | White Matter | 0.31 ± 0.87 | 0.35 ± 0.77 | -0.13 ± 0.91 | 0.22 ± 0.67 |
| White matter R |  | 0.53 ± 0.83 | 0.45 ± 0.46 | -0.05 ± 0.85 | 0.32 ± 067 |
| Cerebellar white matter |  | 1.00 ± 0.40 | 0.84 ± 0.60 | 0.61 ± 0.71 | 0.68 ± 0.50 |
