# Supplementary material for: Alzheimer’s disease pattern derived from relative cerebral flow as an alternative for the metabolic pattern using SSM/PCA
Source: EJNMMI Res. 2022 Jun 23;12:37. doi: 10.1186/s13550-022-00909-8 (PMC9226207; doi:10.1186/s13550-022-00909-8)
Supplement: Supplementary file 2 — Additional file 2. Table S2: Number of subjects (N), mean, standard deviation (SD), standard error of the mean (SEM), and range of scores per diagnosis for each type of image used in the analysis. [file 13550_2022_909_MOESM2_ESM.docx]

**Supplementary Table S2.** Number of subjects (N), mean, standard deviation (SD), standard error of the mean (SEM), and range of scores per diagnosis for each type of image used in the analysis.

| **Image** | **Diagnosis** | **N** | **Mean** | **SD** | **SEM** | **Range** |
| --- | --- | --- | --- | --- | --- | --- |
| FDG | AD | 15 | 2.31 | 1.29 | 0.33 | [-0.88, 4.91] |
|  | MCI+ | 11 | 0.83 | 0.62 | 0.19 | [-0.42, 1.60] |
|  | MCI- | 10 | 0.77 | 1.76 | 0.59 | [-2.68, 3.69] |
|  | HC | 16 | 0.00 | 1.00 | 0.25 | [-1.74, 2.02] |
| *R*_1_ | AD | 15 | 1.62 | 1.00 | 0.26 | [-1.80, 2.72] |
|  | MCI+ | 11 | 0.50 | 0.57 | 0.17 | [-0.39, 1.28] |
|  | MCI- | 10 | 0.24 | 1.05 | 0.35 | [-1.07, 2.46] |
|  | HC | 16 | 0.00 | 1.00 | 0.25 | [-1.81, 2.56] |
| ePIB(20-130s) | AD | 15 | 1.11 | 1.21 | 0.31 | [-2.59, 2.63] |
|  | MCI+ | 11 | 0.547 | 0.49 | 0.15 | [-0.34, 1.34] |
|  | MCI- | 10 | 0.184 | 0.52 | 0.17 | [-0.66, 0.98] |
|  | HC | 16 | 0.00 | 1.00 | 0.25 | [-2.17, 2.89] |
| ePIB(1-8min) | AD | 15 | 0.77 | 0.69 | 0.18 | [-1.29, 2.09] |
|  | MCI+ | 11 | 0.48 | 0.72 | 0.22 | [-0.94, 1.52] |
|  | MCI- | 10 | 0.45 | 0.55 | 0.25 | [-0.36, 1.28] |
|  | HC | 16 | 0.00 | 1.00 | 0.18 | [-2.07, 1.51] |
